# Supplementary material for: The ability of human TIM1 to bind phosphatidylethanolamine enhances viral uptake and efferocytosis compared to rhesus and mouse orthologs
Source: J Virol. 2024 Oct 30;98(11):e01649-24. doi: 10.1128/jvi.01649-24 (PMC11575270; doi:10.1128/jvi.01649-24)
Supplement: Supplemental figures — Figures S1 to S6. [file jvi.01649-24-s0001.pdf]

SUPPLEMENTAL FIGURES

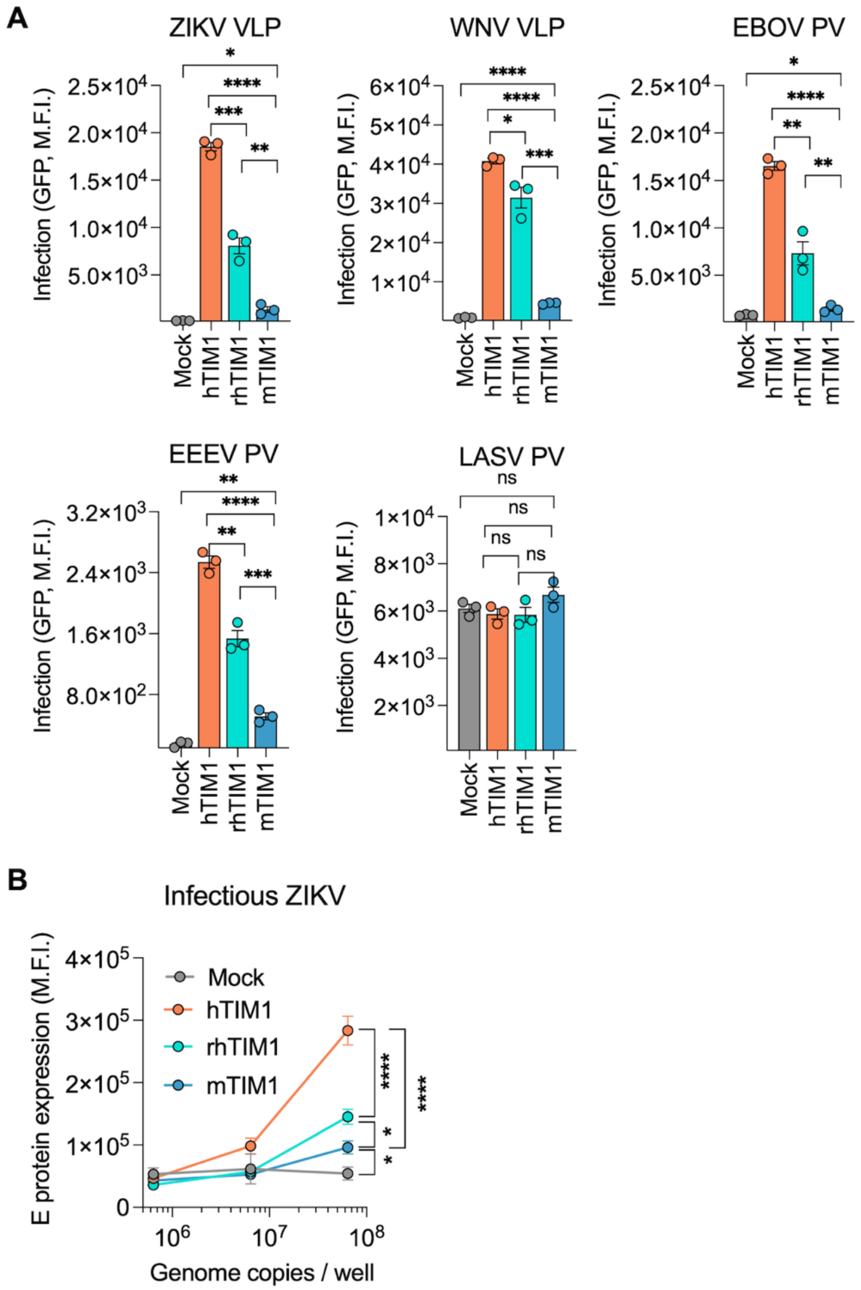

Fig. S1 (related to Fig. 1). mTIM1 and rhTIM1 are not as efficient as hTIM1 in mediating virus infection. (A and B) *The same data presented in Fig. 1C and 1D are analyzed by mean fluorescence intensity (M.F.I.) of GFP and sown here as* ***A*** *and* ***B****, respectively. Statistical significance was analyzed by unpaired t test. *p<0.05, **p < 0.01, ***p < 0.001, and ****p < 0.0001; ns, not significant.*

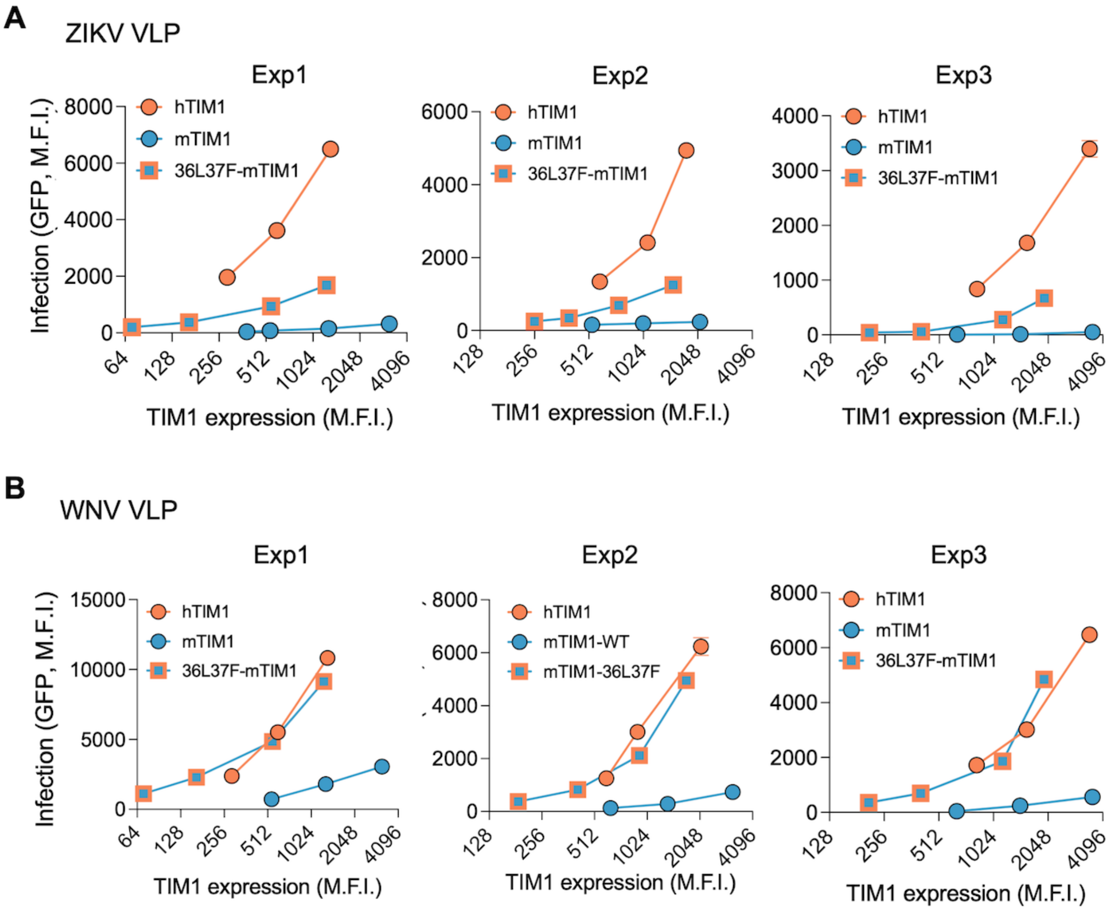

Fig. S2 (Related to Fig. 6). A PE-binding mutant of mTIM1 more efficiently mediates virus entry. *The same data presented in Fig. 6A and B are analyzed by mean fluorescence intensity (M.F.I.) and shown here as* ***A*** *and* ***B****, respectively.*

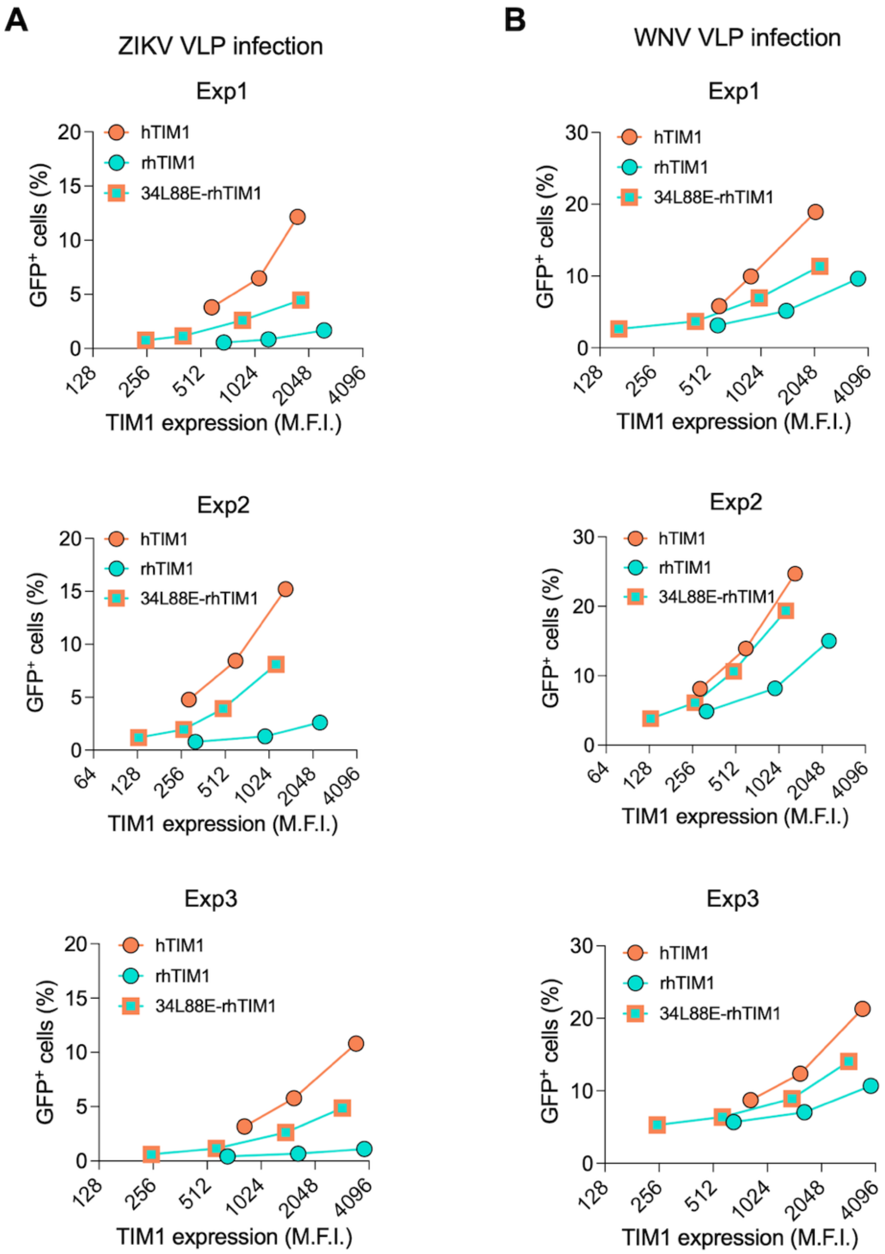

Fig. S3 (Related to Fig. 6). A PE-binding mutant of rhTIM1 more efficiently mediates virus entry. *Three independent infection experiments using ZIKV VLP* ***(A)*** *or WNV VLP* ***(B)*** *were performed and analyzed in the same way as described in Fig. 6A and B except that PE-binding rhTIM1 mutant (34L88E-rhTIM1) was compared to WT-rhTIM1 and hTIM1. Infected cells were analyzed by % GFP positive cells. Three sets of data from three independent experiments are shown.*

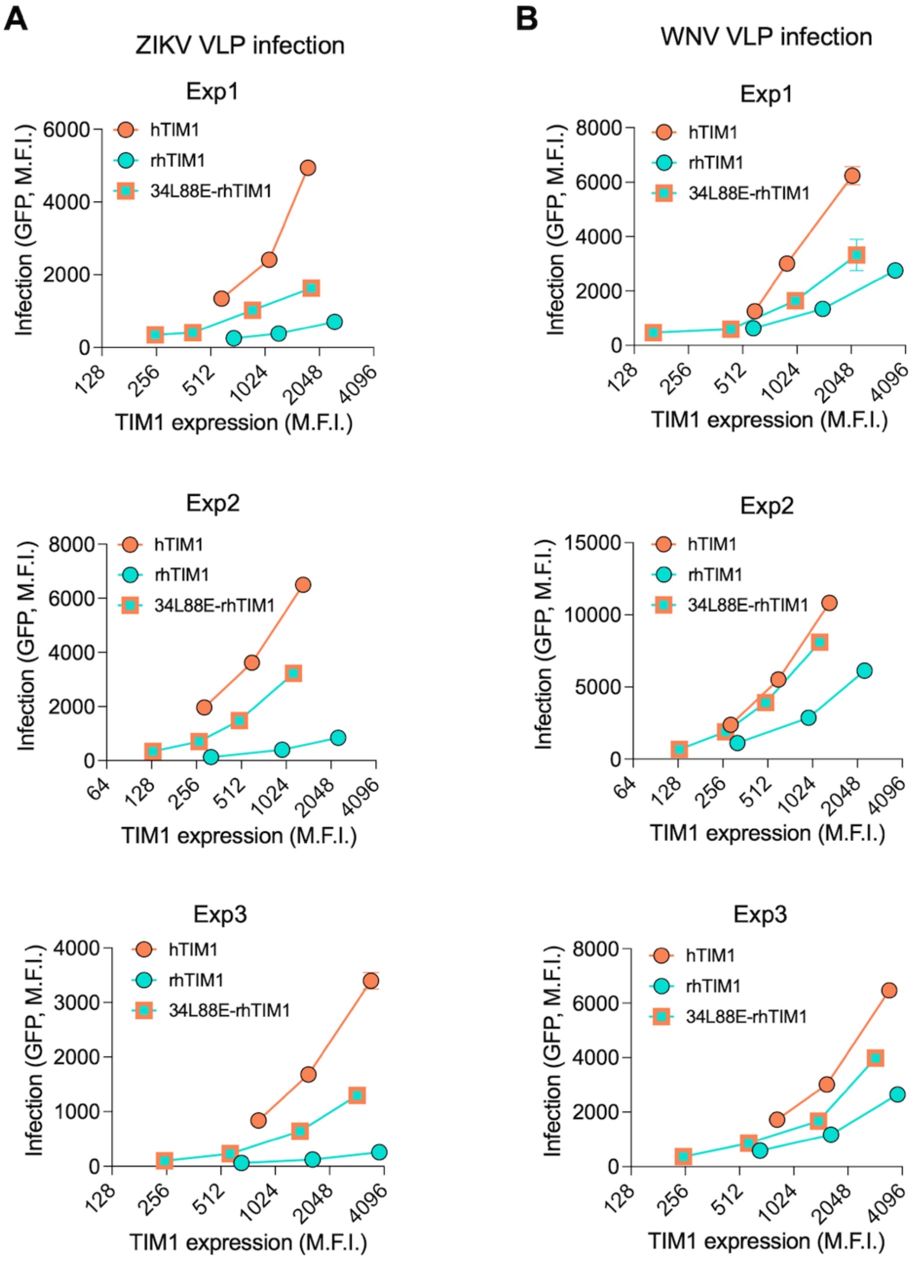

Fig. S4 (Related to Fig. 6). A PE-binding mutant of rhTIM1 more efficiently ***mediates virus entry.*** *The same infection data shown in Fig. S3 were analyzed by mean fluorescence intensity (M.F.I.).*

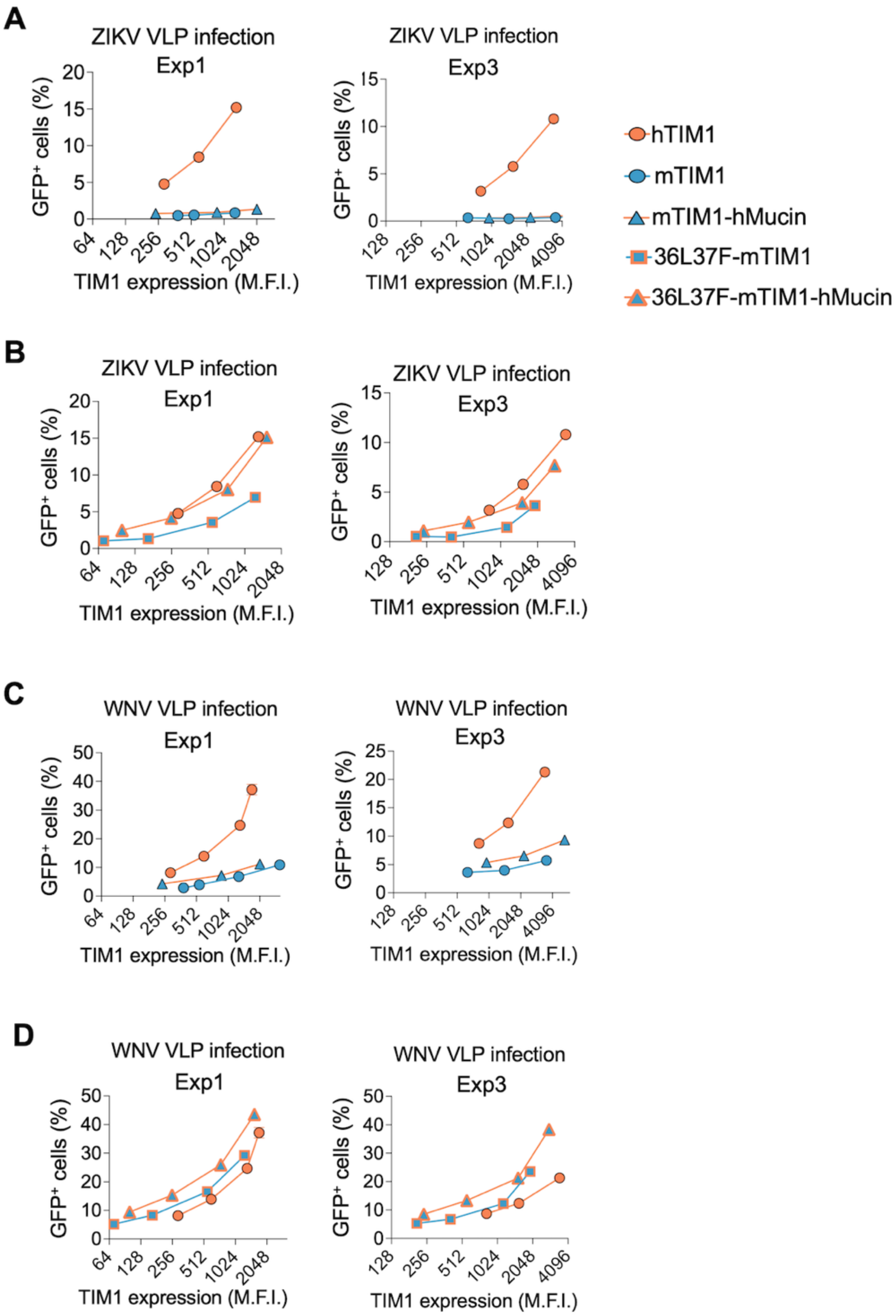

Fig. S5 (Related to Fig. 8). hTIM1 mucin domain cooperates with the PE-binding ability of the head domain in mediating virus entry. (A-D) *Two additional infection experiments for ZIKV VLP (A and B) and WNV VLP (C and D) mediated by mTIM1 with or without the hTIM1 mucin domain (A and C) or by 36L37F-mTIM1 with or without the hTIM1 mucin domain (B and D). Experiments were performed and analyzed as described for Fig. 8B-E.*

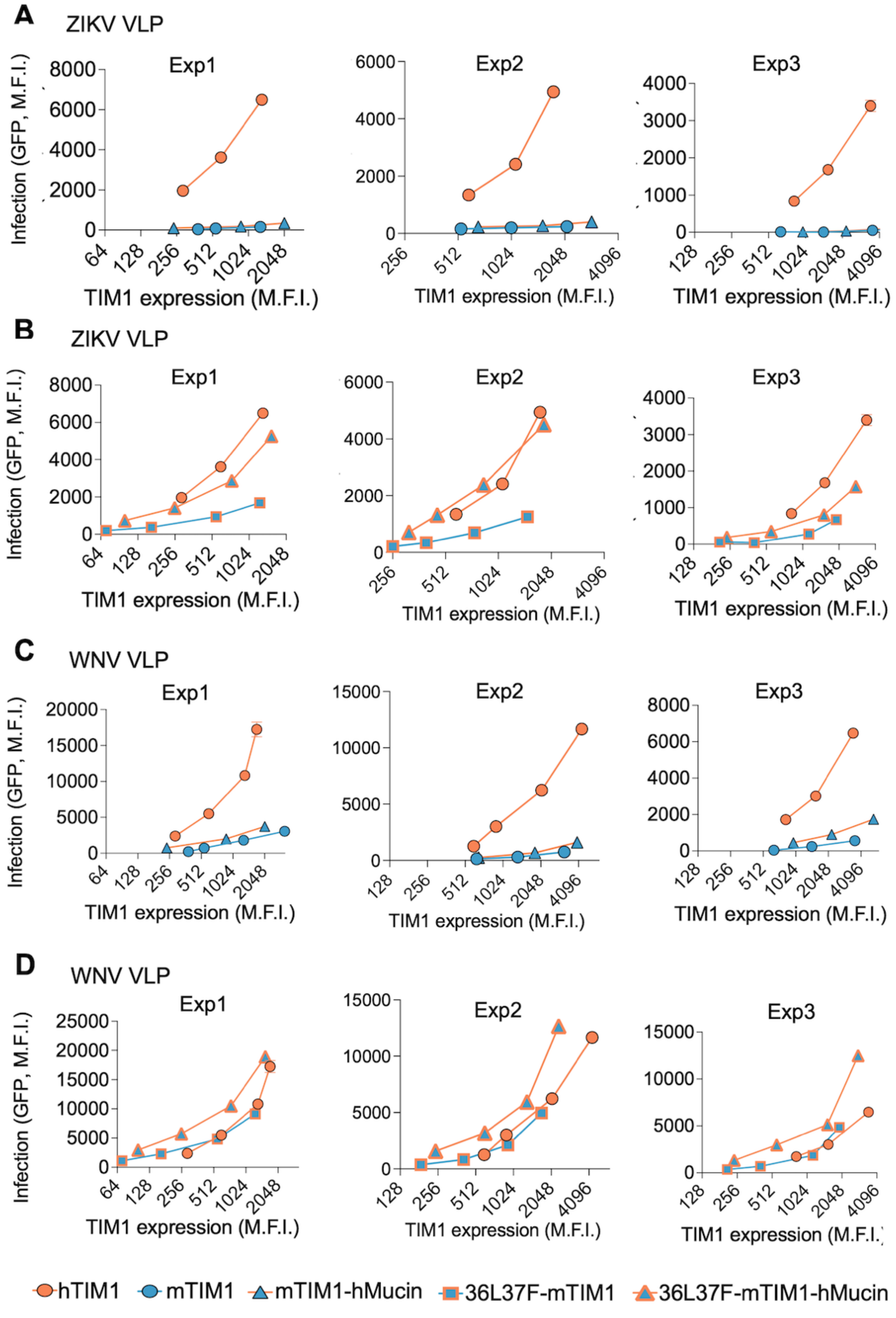

Fig. S6 (Related to Fig. 8). hTIM1 mucin domain cooperates with the PE-binding ability of the head domain in mediating virus entry. *The same data shown in Fig. 8B-E and S5 were analyzed by mean fluorescence intensity (M.F.I.) of GFP. Infection of*

ZIKV VLP (A and B) and WNV VLP (C and D) mediated by mTIM1 with or without the hTIM1 mucin domain (A and C) or by 36L37F-mTIM1 with or without the hTIM1 mucin domain (B and D).
